# Reading in Children Who Survived Cerebellar Tumors: Evidence from Eye Movements

**DOI:** 10.3390/vision6010010

**Published:** 2022-02-06

**Authors:** Sofia Mironets, Marina Shurupova, Anna Dreneva

**Affiliations:** 1 Dmitry Rogachev National Medical Research Center of Pediatric Hematology, Oncology, and Immunology, Neurocognitive Laboratory, 117997 Moscow, Russia; sofiamironets@gmail.com (S.M.); shurupova@fccps.ru (M.S.); 2 Research Institute for Brain Development and Peak Performance, Peoples Friendship University of Russia, 117198 Moscow, Russia; 3 Department of Neurobiology, Faculty of Biology, Lomonosov Moscow State, 119234 Moscow, Russia; 4 Department of Rehabilitation, Federal Center of Brain and Neurotechnologies, 117513 Moscow, Russia; 5 Faculty of Psychology, Lomonosov Moscow State University, 125009 Moscow, Russia; 6 Research Institute for Healthcare Organization and Medical Management of Moscow Healthcare Department, 115088 Moscow, Russia

**Keywords:** pediatric cerebellar tumor, cancer survivors, eye movements, reading

## Abstract

Cerebellar tumors often affect the eye movement centers located in vermis, negatively affecting cognitive development and learning abilities in children. Previous research has established that patients who survived cerebellar tumors tend to demonstrate various saccadic impairments (e.g., hypermetria) and poor gaze stability as compared to healthy controls. The aim of the current study was to evaluate the influence of oculomotor deficits in such patients on reading parameters. A total of 112 children (8–17 y.o.), 65 of whom survived cerebellar tumors, participated in the study. The study design included several oculomotor and reading tasks. Eye movements were recorded every 1/60 s monocularly with an Arrington eye tracker. We observed profound reading impairments in the patients as compared to healthy children, including longer reading time, greater numbers of fixations and regressive saccades, and longer fixation durations. We also found significant correlations between changes in basic oculomotor functions and reading parameters. The patients also demonstrated gaze fixation instability, large number of fixations, and long scanpath reflecting the return of the gaze to the already counted objects. Thus, oculomotor changes caused by cerebellar tumor and its treatment led to disturbances in such neurocognitive activity as reading. Our findings emphasize the necessity of considering these deficits in cerebellar tumor survivors when designing rehabilitation protocols.

## 1. Introduction

Progress in diagnostics and treatment in neuro-oncology has led to a substantial increase of survival rates [1]. However, early and late disturbances in brain functions, resulting from both the disease and the treatment, present a considerable challenge to normal development in children.

Brain tumors are the second most common pathology among children and adolescents. Posterior fossa tumors comprise 60% of them [2], arising in the posterior fossa or the cerebellum and gradually progressing through cerebrospinal pathways while forming new tumors along the ventricles [3].

Since the cerebellum is involved in motor functioning [4,5], its damage often causes motor deficits affecting all levels, including the lowest level of eye movements [6], the latter manifesting in hypometric and hypermetric saccades, nystagmus, periodic alternating nystagmus, failure to suppress the vestibulo-ocular reflex, and so on [7,8,9]. The cerebellum plays an important role in controlling saccadic eye movements and gaze fixation [9,10].

Critical cerebellar structures involved in eye movement control are the vestibulocerebellum (flocculus, paraflocculus, nodulus, uvula, tonsil) and oculomotor cerebellum (vermal lobules V-VII, fastigial nuclei, ansiform lobe—crus I and crus II) [4]. The role of the oculomotor cerebellum in saccadic amplitude control [4] was determined in primate models of local cerebellar lesions [11,12] and studies of cerebellar lesions of different etiologies in humans [7,13,14,15], demonstrating the resulting dysmetric saccades.

The vestibulocerebellum is involved in gaze-holding control, vestibulo-ocular reflex and, partly, in smooth pursuit [4,9]. Its lesions cause gaze fixation impairments: spontaneous, downbeat, and alternative nystagmus; intrusive micro- and macrosaccades; and post-saccadic drift [16,17].

These and other abnormalities in eye movement control may consequently disturb reading, which plays a most crucial part in knowledge and skills acquisition in children and adolescents [18]. Reading is a complex neurocognitive process consisting in shifting gaze with saccades so that fovea focuses on one consecutive word at a time. It requires precisely controlled oculomotor actions for correct information extraction and processing. The areas involved in reading are widely distributed in the brain, and over the past decades, clinical, neuroanatomical, and functional neuroimaging studies have established the involvement of the cerebellum in this process as well [19]. This close link between the ability to read and learn and other implicit learning processes is rooted in the automatized mechanisms comprising reading: phonological processing; automatizing basic auditory and articulatory skills, which are partly mediated by the cerebellum [20]; the reciprocal connections between them; as well as progress tracking while reading [21,22].

The cerebellum has been demonstrated to be involved not only in motor functions control, but cognitive as well [23]. Injury near or inside the cerebellum causes a cerebellar cognitive affective syndrome [8]. The syndrome is characterized by executive function deterioration manifesting in decreased planning ability, working memory, and verbal fluency [24]; impaired spatial cognition; personality changes; and linguistic difficulties including agrammatism, dysprosodia, and mild anomia [8]. Moreover, the cerebellum was revealed to be one of the most consistent locations in which structural differences between dyslexic and control participants are found in imaging studies [25].

The first demonstration of cerebellum involvement in language deficits in children was provided by Daly and Love in 1958 with their account of “the posterior fossa syndrome”. Since then, several studies have found various language deficits caused by tumors in children [26,27,28]. Other studies in children with cerebellar tumors show that right cerebellar tumors are connected with poor literacy and verbal functioning as compared to samples with left cerebellar tumors and spatial deficits [29].

As of now, there are many studies on language, reading, or literacy dysfunction, but they mostly comprise such cerebellar disorders as dyslexia, autism spectrum disorder, attention deficit hyperactivity disorder, William syndrome, and so on (for a review, see [30]). However, there is a lack of studies investigating eye movements while reading in children who survived cerebellar tumors.

The present study is aimed at analyzing in detail the eye movement parameters during reading in children and adolescents who survived cerebellar tumors as compared to healthy controls. Furthermore, we plan to investigate the connection between basic oculomotor parameters and reading performance.

## 2. Materials and Methods

### 2.1. Participants

A total of 112 children (8–17 years old) participated in the study, 65 of whom survived cerebellar tumors, while the other 47 were healthy controls. Participants were Russian native speakers. All participants had normal vision and completed at least 2nd grade of school education. The participants in the control group had no history of neurologic or ophthalmic symptoms or reading difficulties. The exclusion criteria for both groups of patients and healthy controls included epilepsy, poor visual acuity, inability to read from a computer monitor, inability to hold head and posture well to perform the tasks, difficulty to obtain adequate recordings due to corrective lenses, visual field defects, other neurological or ophthalmic issues, and other causes of reading difficulties. All participants were permitted to wear their own corrective lenses or contacts during the study if they did not interfere with the recording quality.

The patients had the following diagnoses: medulloblastoma (36 children), astrocytoma (20 children), and ependymoma (9 children). Children were in remission (i.e., after completion of treatment, including chemotherapy) for a period of 3 to 158 months (M = 41.25, SD = 33.79). The age at disease onset ranged from 3 months to 16 years (M = 7.83, SD = 3.46 years).

All participants older than 15 and the legal representatives of younger children gave written informed consent before the initial tests. The experiment was approved by the Ethics Committee of Dmitry Rogachev National Medical Research Center of Pediatric Hematology, Oncology and Immunology (protocol number 8e/13-17 of 27.10.2017) and was run according to the Declaration of Helsinki.

### 2.2. Apparatus

The eye movements were recorded every 1/60 s monocularly with an Arrington eye tracking system (Arrington Research Inc., Scottsdale, Arizona, USA) (for details, see [7]). The system has a chin support for subjects. Center of vision is detected by locating the center of the pupil. Calibration was performed using the standard nine-point algorithm. Subjects sat approximately 60 cm in front of a computer monitor (Samsung, 23″, resolution 1920 × 1080 pixels). The images were presented on a monitor using built-in ViewPoint EyeTracker^®^ 2.9.2.5.

### 2.3. Stimuli and Procedure

#### 2.3.1. Saccadic Tasks

Experiments used two oculomotor tests run with originally developed stimuli, as well as the material based on the relevant literature and adapted for the experimental conditions [7]. Two tests were performed: (i) A fixation task in which the subject was required to look at a target—a green circle (diameter ~1°) sequentially presented at eccentric (15° rightward and leftward, 8° upward and downward) positions, each eccentric position being sustained for 20 s. Gaze-holding score was assessed for each circle by approximating the gaze position coordinates by the least squares method to obtain the best correspondence using the fit function [31]. The areas of the obtained ellipses, reflecting the spread of gaze position coordinates, were determined for each of the four circle positions in each subject. Areas were expressed in square visual degrees (sq. deg.). Artifactual values due to incorrect detection of the pupil in some subjects were culled. For further analysis, the average gaze-holding score was calculated for every participant. (ii) A “visual search task”. Ten black circles (diameter ~1°) were presented on the monitor distributed pseudorandomly over the screen. Subjects were instructed to count silently the number of points and give a verbal response. The results of this task were used to analyze the performance time, the number of fixations, their durations, the total length of the scanning trajectory, and the saccade amplitude. The task performance time was considered as the time necessary to give a response of the number of points. The total scanning trajectory was computed as the sum of the amplitudes of all saccades. Analysis of fixations excluded fixations of duration < 80 msec.

#### 2.3.2. Reading Task

We used seven literally simple texts extracted from the Russian language textbook used by the primary school under the Russian Ministry of Education. Each text contained a paragraph of 5 to 7 lines, 36–65 words, and a maximum of 53 characters per line plus spaces/line in Arial size 48. The automated readability index (ARI) of texts varied from 0.06 to 10.77. The texts did not differ in inter-letter space. The children read passages presented on the screen. The two questions about each text were asked to ensure participants understood their meaning. Participants were asked to read silently as natural reading without making errors. Then, we included the mean measures of all eye movement parameters in all texts into the analysis. We considered the following parameters: (a) total reading time; (b) reading word per second; (c) total number of fixations; (d) number of fixations per word; (e) number of fixations per row; (f) average fixation duration; (g) percentage of saccadic regressions; (h) progressive saccadic amplitude; and (i) coefficient of variance (CV) of progressive saccade amplitude.

### 2.4. Statistical Analysis

Gaze position coordinates were determined using ViewPoint EyeTracker^®^ 2.9.2.5 software (Arrington Research Inc.); events (fixations and saccades) were extracted using DataAnalysis software in the Arrington Research program bundle. Ellipse areas, saccade amplitudes, fixation durations, and their numbers were processed statistically in Statistica 13.3 (TIBCO Software Inc., Palo Alto, California, USA).

The Kolmogorov–Smirnov normality test showed non-normal distribution of the data (*p* > 0.05). Hence, the eye movement behaviors in reading were compared using the Mann–Whitney U-Test with the Bonferroni correction (significance level: 0.002). We also performed the analysis of correlations between oculomotor and reading measures using Spearman’s rank correlation coefficient. The statistical significance was set at *p* < 0.05.

## 3. Results

The group of cerebellar tumor survivors included sixty-five children (aged 12.35 ± 2.44) (52% female). The control group included forty-seven healthy children (aged 11.87 ± 2.41) (57% female). In 90% of cases, participants gave the right answer to the questions about the texts, which allows us to consider a high level of understanding of the text.

### 3.1. Comparing Eye Movements during Reading Texts in the Patient Group and Control Group

Firstly, significant differences between patient and control groups were found in seven reading measures, with lower performance seen in the former (Table 1).

Patients had longer reading time (U = 1017, *p* = 0.003, ES = 0.28) and read less words per second than the control group (U = 971, *p* = 0.001, ES = 0.31). The patients also showed a significantly larger total number of fixations (U = 1124, *p* = 0.018, ES = 0.225), as well as a larger number of fixations per word (U= 1033, *p* = 0.004, ES = 0.275), and a longer average fixation duration (U = 1109, *p* = 0.014, ES = 0.233). The patients also had a significantly higher percentage of saccadic regressions (U = 910, *p* < 0.001, ES = 0.286). The large variability of progressive saccades was represented in an increased CV of progressive saccade amplitude (U = 987, *p* = 0.001, ES = 0.301). Surprisingly, groups were not significantly different according to the progressive saccadic amplitude (U = 1262, *p* = 0.118, ES = 0.148), and the number of fixations per row did not reach a significant level (U = 1196, *p* = 0.051, ES = 0.184). Thus, most of the reading measures were poorer in the patient group.

### 3.2. Comparing Eye Movements during Oculomotor Tasks in the Patient Group and Control Group

Since the reading task showed a pronounced difference between the patients and the control group, we further analyzed the oculography data in oculomotor tasks to determine why patients read slower. As compared with the controls, patients had a significantly greater gaze-holding score (U = 600, *p* < 0.001, ES = 6.34) (Table 2), which indicated the impaired gaze-holding process in these children. We observed the same results in the visual search task: the patients showed a large number of fixations (U = 783, *p* < 0.001, ES = 7.10) and longer scanpath lengths (U = 880, *p* = 0.029, ES = 83.638), reflecting the recounting of objects which had already been counted. The time of performing visual search task (U = 633, *p* < 0.001, ES = 3.882), number of fixations, and scanpath lengths were significantly different, with no differences in the mean fixation duration (U = 1001, *p* = 0.184, ES = 0.058) or mean saccade amplitude (U = 963, *p* = 0.110, ES = 1.922) (Table 2).

### 3.3. Analysis of the Correlation between Eye Movements during Reading and Two Oculomotor Tests in Cerebellar Tumor Survivors

We examined the correlation between the eye movement measures during reading and two core eye movement tests in children who survived cerebellar tumors (Table 3). We observed significant correlations between gaze-holding score and the reading measures: the more unstable gaze holding was, the more reading time (r = 0.297, *p* = 0.018) and fewer words the patients read in one second (r = −0.321, *p* = 0.010). We found a positive correlation between the gaze-holding stability and the percentage of saccadic regressions (r = 0.352, *p* = 0.005), as well as between the number of fixations per word positively and the gaze-holding score (r = 0.249, *p* = 0.049).

In the visual search task, the time of performing negatively correlated with the number of words per second (r = −0.257, *p* = 0.046). Moreover, the number of words per second negatively correlated with scanpath length (r = −0.257, *p* = 0.045) and the number of fixations in the visual search task (r = −0.319, *p* = 0.012). 

There was also a positive correlation between the number of fixations in the visual search task and reading measures, including the total reading time (r = 0.311, *p* = 0.015), the total number of fixations (r = 0.268, *p* = 0.037), and the average fixation duration (r = 0.253, *p* = 0.049). Similarly, the number of fixations in the visual search task positively correlated with the number of fixations per line (r = 0.264, *p* = 0.040) and the number of fixations per word (r = 0.267, *p* = 0.038).

### 3.4. Analysis of the Impact of Histology and Age at Disease Onset on the Eye Movements during Reading in Cerebellar Tumor Survivors

The comparison of eye movement measures during reading between the patients after malignant (medulloblastoma and ependymoma) and benign (astrocytoma) tumors showed no significant difference (all *p* > 0.1, M-W test).

We revealed several correlations between the eye movements during reading and age at disease onset (Table 3). In the patient group, age at disease onset significantly correlated with total reading time (r = −0.360, *p* =0.003), number of reading words per second (r = 0.380, *p* = 0.002), number of fixations per word (r = −0.307, *p* = 0.013), number of fixations per line (r = −0.354, *p* = 0.004), number of fixations per word (r = −0.343, *p* = 0.005), fixation duration (r = −0.391, *p* = 0.001), amplitude of progressive saccades (r = 0.451, *p* < 0.001), and CV of amplitude progressive saccades (r = −0.288, *p* = 0.020). The correlation with the percentage of regressive saccades was not significant (r = 0.010, *p* = 0.935).

### 3.5. Analysis of the Correlation between Eye Movements during Reading and Two Oculomotor Tests in the Control Group

The same analysis of correlations was performed with the results of the control group.

We revealed the correlation between the measures of the “gaze-holding task” and reading parameters: the more stable gaze holding was in stimulus, the less time was needed for reading (r = 0.363, *p* = 0.030), and accordingly, the more words the child read in a period of time (r = −0.376, *p* = 0.025). The more stable the gaze holding was, the bigger the amplitude of progressive saccades was when reading (r = −0.336, *p* = 0.046).

There were positive correlations between the gaze-holding score and the total number of fixations (r = 0.399, *p* = 0.016), number of fixations per line (r = 0.377, *p* = 0.024), and number of fixations per word (r = 0.410, *p* = 0.014).

Correlations were found between the measures of the “visual search task” and reading parameters: the less time spent in the visual search task, the higher was the speed of reading (r = 0.396, *p* = 0.014) and more words per second were read (r = −0.431, *p* = 0.007). Similarly, the longer time in visual search task was correlated with more fixations (r = 0.443, *p* = 0.005), more fixations per line (r = 0.452, *p*= 0.005), and more fixations per word (r = 0.440, *p*= 0.006) during reading. The longer time in the visual search task positively correlated with the percentage of saccadic regressions during reading (r = 0.391, *p* = 0.016).

There were positive correlations between the mean fixation duration in the visual search task and reading measures, including the total reading time (r = 0.382, *p* = 0.017), the total number of fixations (r = 0.419, *p* = 0.008), number of fixations per line (r = 0.420, *p* < 0.008), number of fixations per word (r = 0.400, *p* = 0.012), and average fixation duration (r = 0.379, *p* = 0.018). There was a negative correlation with words per second in reading (r = −0.388, *p* = 0.015).

The analysis of age-related correlations of the eye movement parameters during reading in healthy children showed an increase in speed reading (r = −0.568, *p* < 0.001), a decrease in the total number of fixations (r = −0.510, *p* < 0.001), the number of fixations per word (r = −0.545, *p* < 0.001), and the number of fixations per row (r = −0.506, *p* < 0.001). Moreover, with age, healthy children showed a decrease in the percentage of regressive saccades (r = −0.351, *p* = 0.015) and an increase in the amplitude of progressive saccades (r = 0.303, *p* = 0.038). In patients, such dynamics of changes in speed reading, a decrease in the number of fixations, and saccade amplitudes with age were not revealed. 

### 3.6. Exploring Eye Movements Peculiarities in Reading and Oculomotor Measures Using Correlational Analysis in the Patient Group and the Control Group

In all participants, gaze-holding score significantly correlated with the total reading time, the reading word per second, and the number of fixations per word. The gaze holding score had a positive correlation with the percentage of saccadic regressions in the patient group but not in the control. The gaze-holding score correlated with the total number of fixations and the number of fixations per row reached the level of significance in the control group but not in the patients. The gaze-holding score had a negative correlation with the progressive saccadic amplitude in the control group and had no correlation in the patient one.

There were positive correlations in the visual search task (time) and the total reading time, the total number of fixations and fixations per word and row, as well as the progressive saccadic amplitude during reading texts in the control group but not in patients. There was a negative correlation between the visual search task (time) and the number of words per second in reading in each group of participants.

The number of fixations in the visual search task correlated with the total reading time and reading word per second.

We can observe the same correlations between the number of fixations in the visual search task and fixation parameters in reading (the total number of fixations; the number of fixations per word; the number of fixations per row; average fixation duration). These are correlations typical for the patient group but not for control.

The mean fixation duration in the visual search task correlated with reading parameters: the total reading time; reading word per second; the total number of fixations; the number of fixations per word; the number of fixations per row; and average fixation duration in the control group but not in the patient group.

The scanpath lengths negatively correlated with the number of words per second in the patient group but there was no such correlation in the control group.

## 4. Discussion

In the current study, we measured quantitative oculomotor and reading indicators in a fairly large sample of pediatric cerebellar tumor survivors. Our main finding is the negative effect of cerebellar damage due to tumor and its later treatment on oculomotor functioning and reading in these patients.

The analysis of reading performance revealed a wide range of deficits in patients (Table 1): slow reading, larger total number of fixations and fixations per word, longer fixation duration, increased percentage of saccadic regressions, and larger variability of progressive saccades. Very few studies conducted on patients with similar condition have assessed their reading skills [29,32,33], while to our knowledge, there are no studies in which oculomotor parameters changes were described. Oculomotor parameters while reading are thought to reflect reading skills level [34,35,36]; therefore, we assume these skills to be impaired in our patients as a result of existing cerebellar dysfunction.

Thus, for further clarification of the cerebellar contribution to the oculomotor component of reading, we analyzed basic oculomotor activity such as gaze holding and simple visual search. Comparison of fixation stability (Table 2) showed that cerebellar patients experience persistent difficulties, manifesting in low-amplitude intrusive saccades, nystagmus, and high-amplitude contextually inappropriate saccades, which indicate attention disruption as evaluated by investigators during the experiment. These results are consistent with previous findings [7] and studies on other cerebellar pathologies [17,37]. Gaze fixation instability arises from lesions of cerebellar lobules—the flocculus and paraflocculus—which project to the brainstem saccade generator [9,16]. Attention disruption is also a well-known symptom of cerebellar cognitive-affective syndrome [8]. The analysis of visual search performance (Table 2) revealed poorer results in patients compared to the control group: longer time of task execution, larger number of fixations, and longer scanpath lengths. These impairments account for weak visuospatial organization and integration in cerebellar patients that was emphasized in previous studies [8,38]. It is caused by a malfunction in the cerebellar-cortical interaction carried out by the cortical-ponto-cerebellar afferent pathways and cerebellar-thalamo-cortical efferent pathways [39].

Finally, we investigated the relationships between parameters of core oculomotor activity and reading in patients and controls (Table 3). We emphasize the undertaken correlation analysis is exploratory rather than confirmatory. We found that gaze fixation stability correlates with several reading parameters in both groups—total reading time, the reading word per second, and the number of fixations per word. The most probable conclusion is that more stable fixations allow for more successful textual information extraction and further processing. Since fixation duration is mainly associated with the processes of integration and analysis of visual input [40,41], our findings contribute to the idea that disrupted fixation control results in greater number of repetitions and re-reading, which, in turn, require new fixations, further slowing overall reading process. Moreover, we found a correlation between gaze holding and percentage of regressive saccades. Regressive saccades are known to occur due to misunderstanding the text [42] or due to oculomotor mistakes [43] resulting from incorrect oculomotor program or a saccade drift. This finding also contributes to the hypothesis that unstable gaze fixation is directly related to difficulties in text processing. In the control group, in addition to correlations with other indicators of fixation number, we also observed that gaze fixation stability is directly correlated with amplitude of progressive saccades when reading. This correlation confirms previous results [35,36] associating more developed oculomotor reactions with more skilled reading.

Saccadic coordination when performing visual search task develops along with reading skills [36]. Correlation analysis demonstrated that different parameters are associated with reading performance in control and cerebellar groups (Table 3). In cerebellar patients the number of fixations was directly correlated with reading efficiency, while in control patients the time of the task completion and mean fixation duration were such correlates. One possible explanation could be that cerebellar patients had pronounced difficulties in executing precise patterns of eye movement, so a greater number of fixations appeared due to the necessity to execute a greater number of corrective saccades. Fixation instability from square wave jerks, saccadic oscillations, ocular flutter, nystagmus, and micro-saccades may also contribute to slower searching, causing time losses while executing these eye movements. We observed a large number of non-significant fixations during reading tasks, which were excluded from the analysis at the preprocessing stage. These abnormalities lead to more frequent and abnormal saccades and fixations during reading as well as other visual tasks [44], while healthy children executed effective eye movements, thus reducing performance time for both searching and reading tasks. Correlation between performance time for searching task and reading efficiency underline their similarity. Previous studies showed that fewer and quicker fixations were found among children with higher reading level [45]. However, it remains unclear why there was no significant correlation between the fixation duration and reading parameters in the control group.

We also correlated the age of participants to the reading parameters and found a clear dependence on age (except CV of progressive saccades) in the control group, while there was no correlation in the cerebellar group (Table 3). The improvement of reading skills with age has been reported in numerous studies [35,36,46]. On one hand, saccadic cortical areas (frontal and parietal cortex) are less active in young children than in adolescents and adults [47], pointing to maturation of these areas with age. The same dependence on age is known for structures involved in linguistic processing. On the other hand, Mariën et al. [48] in their consensus paper summarized and emphasized the cerebellar functions that might be responsible for reading formation during typical development.

Absence of correlation between age and reading efficiency in the cerebellar group might arise from them having different saccadic cortical and cerebellar structures development levels due to the different age of the disease onset. Clinical studies demonstrated early age of disease onset to be a negative prognostic factor, since most of the structures and afferent-efferent systems have not yet been formed and may not be formed properly as a result of the ongoing pathological process [49,50]. We also found an inverse correlation between age at the disease onset and the extent of eye movement deficits (Table 3). Therefore, comparing these results with the age-dependency of these parameters in the control group, we should emphasize the important role of the early rehabilitation process in young cerebellar cancer survivors.

Although aggressiveness of the treatment, and, therefore, damage to motor and cognitive functions, depends on the malignancy of the tumor, we found no significant difference in eye movement parameters while reading between malignant (medulloblastoma and ependymoma) and benign (astrocytoma) tumor survivors. Perhaps, the illness and its surgical treatment are sufficient for the development of oculomotor disturbances leading to reading impairments. Future research should focus on the influence of tumor location and the extent of the damage to fine cognitive skills.

One of our most intriguing results is the failure of differences between patients and controls in the amplitude of progressive saccades (Table 1). Lesions of the cerebellum in V–VII vermal lobules and fastigial nucleus lead to impaired saccade amplitude calculation (hypermetry or hypometry) [4,9], and in our previous work we found hypermetric saccades in children who survived cerebellar tumors [7]. Thus, we would expect an increased saccade amplitude in patients while reading. At the same time, increased saccade amplitude, as well as decreased amount of fixations and their duration, are characteristic for skilled reading [34,35,36]. We found a significant difference in CV of progressive saccade amplitude between the two groups. The cerebellar group had greater variability in saccade amplitude due to a greater number of dysmetric saccades (both hypo- and hypermetric) caused by inappropriate saccade programming, while mean saccade amplitude did not differ from the mean for the control group, since small and large amplitudes balance each other. We also found an increase in the fixation duration resulting from an increase in the proportion of dysmetric saccades [41].

Thus, in the study we investigated the oculomotor reading deficits caused by cerebellar dysfunction. Nicolson et al. [51], in the “cerebellar deficit hypothesis”, proposed that disturbances of cerebellum development lead to reading and writing impairments resembling dyslexia due to its involvement in automatic action execution. In our study, we did not examine dyslexic children but demonstrated the crucial role of the cerebellum in reading. This process requires fine sensorimotor coordination to place a certain area of text in the foveal vision for detailed processing. Cerebellar lesion results in an inaccurate and inadequate biofeedback and, therefore, impaired automatization.

Moreover, the right lateral posterior cerebellum is connected with the left cerebral hemisphere involved in language processing [19]. Therefore, tumor-related and treatment-related cerebellar lesions could also damage the cerebro-cerebellar projections, resulting in an impairment of phonological and semantic processing. However, here we focus more on oculomotor alteration rather than cerebro-cerebellar interactions because the level of of text comprehension was sufficient in both groups (90% of questions on the text were answered correctly).

Likewise, we do not focus our study on the role of cerebellar feedback loop through thalamus to the cerebral cortex including sensorimotor and associative cortices [8], although the pulvinar nucleus of the thalamus is extensively connected with the prefrontal cortex, sensory cortex, superior colliculus, and amygdala, and plays very important roles in contextual multi-sensory processing [52] that might be important for reading in natural conditions.

Thus, in the present study we demonstrated effects of particular oculomotor disturbances on such a cognitive process as reading. Due to oculomotor errors when reading, children develop their own adaptive reading strategy (e.g., performing a higher number of regressive saccades in patients), allowing them not to miss the meaning of the text, but leading to decrease in reading speed and automatization of reading. According to preliminary results of an investigation on reading texts aloud conducted in our hospital by speech therapists [53], these children exhibit phonetic, intonation, and word substitution errors, as was also shown in [32,54]. Hence, we can see how the direct effects of oculomotor disorders on reading mediates indirect effects in cognitive behavior.

Increase of the survival rate of patients with cerebellar tumors requires development of techniques for reintroducing a large number of them to the educational process. Reading skills are crucial for academic performance and general development in children and adolescents. Our results emphasize the necessity of considering them when designing the rehabilitation protocols.

## 5. Conclusions

To conclude, we investigated objective oculomotor and reading indicators in a fairly large sample of young cerebellar tumor survivors. In the study, we found pronounced reading disturbances among cerebellar patients, such as slow reading, larger total number of fixations and fixations per word, longer fixation duration, increased percentage of saccadic regressions, and larger variability of progressive saccades. We demonstrated the effect of impairments of core oculomotor functions (such as gaze holding and visual search) caused by tumor and its treatment on the reading task through different possible mechanisms such as oculomotor errors and adaptive slowdown strategy. The relations between the oculomotor deficits observed during reading and more general reading-performance measures (phonetic and word substitution errors, comprehension difficulties in limited time, inappropriate intonation) need further investigation. Our findings confirm the core role of the cerebellum in oculomotor activity, as well as in higher cognitive processes such as reading. This should be considered when designing the rehabilitation protocols for pediatric cerebellar tumor survivors.

## Figures and Tables

**Table 1:** The parameters of eye movements during reading, averaged over all seven texts, in the control group (CG) and in the group of children who survived cerebellar tumor (PG). Values in bold indicate statistically significant results.

Parameter	Mean		SD		Median		Min		Max		U	Z	*p*-Value	Effect Size
	CG	PG	CG	PG	CG	PG	CG	PG	CG	PG				
Total reading time	**23.869**	**37.530**	**11.771**	**26.054**	**22.236**	**27.067**	**8.416**	**11.678**	**70.885**	**131.797**	**1017**	**3.007**	**0.003**	**0.284**
Reading word per second	**2.362**	**1.707**	**1.017**	**0.898**	**2.123**	**1.711**	**0.637**	**0.273**	**5.755**	**4.181**	**971**	**−3.278**	**0.001**	**0.310**
Total number of fixations	**73.884**	**96.636**	**20.663**	**54.094**	**74.000**	**82.000**	**40.167**	**48.000**	**142.429**	**385.000**	**1124**	**2.376**	**0.018**	**0.225**
Number of fixations per word	**1.605**	**2.196**	**0.454**	**1.307**	**1.625**	**1.863**	**0.872**	**1.059**	**2.992**	**9.619**	**1033**	**2.913**	**0.004**	**0.275**
Number of fixations per row	13.985	17.481	3.861	8.753	14.083	15.667	7.594	6.450	23.320	60.125	1196	1.952	0.051	0.184
Average fixation duration	**258.767**	**315.325**	**73.760**	**199.395**	**250.485**	**270.214**	**151.635**	**151.831**	**597.755**	**1716.400**	**1109**	**2.464**	**0.014**	**0.233**
Percentage of saccadic regressions	**19.848**	**26.908**	**4.863**	**11.210**	**19.653**	**25.417**	**9.782**	**6.625**	**30.003**	**59.259**	**910**	**3.638**	**0.000**	**0.344**
Progressive saccadic amplitude	4.143	3.890	0.931	0.953	4.296	3.782	2.206	1.920	6.332	6.358	1262	−1.562	0.118	0.148
CV of progressive saccade amplitude	**0.413**	**0.443**	**0.053**	**0.062**	**0.412**	**0.443**	**0.268**	**0.181**	**0.568**	**0.582**	**987**	**3.184**	**0.001**	**0.301**

**Table 2:** The gaze-holding score and parameters of visual search task in the control group (CG) and in the group of children who survived cerebellar tumor (PG). Values in bold indicate statistically significant results.

Parameter	Mean		SD		Median		Min		Max		U	Z	*p*-Value	Effect Size
	CG	PG	CG	PG	CG	PG	CG	PG	CG	PG				
Gaze-holding score average	**2.264**	**9.069**	**2.171**	**13.096**	**1.594**	**4.502**	**0.354**	**0.883**	**11.778**	**63.081**	**600**	**3.881**	**0.000**	**6.340**
Performance time of visual search task	**5.360**	**7.907**	**1.872**	**5.204**	**4.770**	**6.342**	**3.203**	**2.568**	**11.522**	**38.618**	**633**	**3.781**	**0.000**	**3.882**
Scanpath length	**203.242**	**235.031**	**207.286**	**147.392**	**144.361**	**199.491**	**92.788**	**56.085**	**1340.435**	**836.375**	**880**	**2.184**	**0.029**	**83.638**
Number of fixations	**16.103**	**20.164**	**7.279**	**10.594**	**15.000**	**18.000**	**10.000**	**6.000**	**51.000**	**71.000**	**783**	**2.869**	**0.004**	**7.100**
Mean of fixation duration	0.271	0.296	0.072	0.090	0.261	0.284	0.162	0.150	0.430	0.579	1001	1.329	0.184	0.058
Mean saccade amplitude	9.778	8.776	3.820	2.742	9.280	8.410	4.980	3.090	28.520	19.220	963	−1.597	0.110	1.922

**Table 3:** Spearman’s rank correlation between the reading measures and two oculomotor tests. Values in bold are significant at *p* < 0.05.

Parameter	Gaze-Holding Score		Time of Visual Search Task		Scan Path Lengths		Number of Fixations		Mean of Fixation Duration		Saccade Amplitude		Age		Age at Disease Onset
	PG	CG	PG	CG	PG	CG	PG	CG	PG	CG	PG	CG	PG	CG	PG
Total reading time	**0.297**	**0.363**	0.247	**0.396**	0.251	−0.006	**0.311**	0.127	−0.018	**0.382**	0.073	−0.096	−0.232	**−0.568**	**−0.360**
Reading word per second	**−0.321**	**−0.376**	**−0.257**	**−0.431**	**−0.257**	−0.044	**−0.319**	−0.177	0.016	**−0.388**	−0.069	0.071	0.235	**0.599**	**0.380**
Total number of fixations	0.219	**0.399**	0.195	**0.443**	0.210	−0.085	**0.268**	0.115	−0.005	**0.419**	0.109	−0.172	−0.238	**−0.510**	**−0.307**
Number of fixations per word	**0.249**	**0.41**	0.196	**0.44**	0.232	−0.016	**0.267**	0.137	−0.008	**0.400**	0.113	−0.102	−0.233	**−0.545**	**−0.343**
Number of fixations per row	0.200	**0.377**	0.193	**0.452**	0.232	−0.028	**0.264**	0.135	−0.022	**0.42**	0.099	−0.101	−0,225	**−0.506**	**−0.354**
Average fixation duration	0,151	0.246	0.214	0.283	0.234	0.011	**0.253**	0.059	0.026	**0.379**	0.220	−0.052	−0.153	**−0.540**	**−0.391**
Percentage of saccadic regressions	**0.352**	0.194	0.174	**0.391**	0.194	0.313	0.204	0.279	−0.040	0.178	0.039	0.034	−0.043	**−0.351**	0.010
Progressive saccadic amplitude	−0.117	**−0.336**	−0.183	−0.253	−0.129	0.168	−0.190	−0.129	−0.033	−0.205	0.053	0.221	0.180	**0.303**	**0.451**
CV of progressive saccade amplitude	0.135	0.147	0.072	0.047	−0.042	0.124	0.113	0.157	0.065	−0.065	−0.076	0.025	−0.077	−0.083	**−0.288**

## Data Availability

Data are available on request.
